# Patchy Blooms and Multifarious Ecotypes of Labyrinthulomycetes Protists and Their Implication in Vertical Carbon Export in the Pelagic Eastern Indian Ocean

**DOI:** 10.1128/spectrum.00144-22

**Published:** 2022-05-03

**Authors:** Ningdong Xie, Mohan Bai, Lu Liu, Jiaqian Li, Yaodong He, Jackie L. Collier, Dana E. Hunt, Zackary I. Johnson, Nianzhi Jiao, Guangyi Wang

**Affiliations:** a Center for Marine Environmental Ecology, School of Environmental Science and Engineering, Tianjin University, Tianjin, China; b Key Laboratory of Systems Bioengineering (Ministry of Education), Tianjin University, Tianjin, China; c School of Marine and Atmospheric Sciences, Stony Brook University, Stony Brook, New York, USA; d Marine Laboratory, Duke University, Beaufort, North Carolina, USA; e Biology Department, Duke University, Durham, North Carolina, USA; f State Key Laboratory of Marine Environmental Science, College of Ocean and Earth Sciences, Xiamen University, Xiamen, Fujian, China; Nanyang Technological University

**Keywords:** biological pump, carbon cycle, dark ocean, ecotype, heterotrophic protist

## Abstract

Labyrinthulomycetes protists are an important heterotrophic component of microeukaryotes in the world’s oceans, but their distribution patterns and ecological roles are poorly understood in pelagic waters. This study employed flow cytometry and high-throughput sequencing to characterize the abundance, diversity, and community structure of Labyrinthulomycetes in the pelagic Eastern Indian Ocean. The total Labyrinthulomycetes abundance varied much more among stations than did the abundance of prokaryotic plankton, reaching over 1,000 cells mL^−1^ at a few “bloom” stations. The total Labyrinthulomycetes abundance did not decline with depth throughout the whole water column (5 to 2,000 m) like the abundance of prokaryotic plankton did, and the Labyrinthulomycetes average projected biomass over all samples was higher than that of the prokaryotic plankton. However, Labyrinthulomycetes diversity showed obvious vertical variations, with richness, Shannon diversity, and evenness greatest in the upper epipelagic, lower epipelagic, and deep waters, respectively. Many abundant phylotypes were detected across multiple water layers, which aligned with the constant vertical Labyrinthulomycetes biomass, suggesting potential sinking and contribution to the biological pump. Hierarchical clustering revealed distinct ecotypes partitioning by vertical distribution patterns, suggesting their differential roles in the carbon cycle and storage processes. Particularly, most phylotypes showed patchy distributions (occurring in only few samples) as previously found in the coastal waters, but they were less associated with the Labyrinthulomycetes blooms than the prevalent phylotypes. Overall, this study revealed distinct patterns of Labyrinthulomycetes ecotypes and shed light on their importance in the pelagic ocean carbon cycling and sequestration relative to that of the prokaryotic plankton.

**IMPORTANCE** While prokaryotic heterotrophic plankton are well accepted as major players in oceanic carbon cycling, the ecological distributions and functions of their microeukaryotic counterparts in the pelagic ocean remain largely unknown. This study focused on an important group of heterotrophic (mainly osmotrophic) protistan microbes, the Labyrinthulomycetes, whose biomass can surpass that of the prokaryotic plankton in many marine ecosystems, including the bathypelagic ocean. We found patchy horizontal but persistent vertical abundance profiles of the Labyrinthulomycetes protists in the pelagic waters of the Eastern Indian Ocean, which were distinct from the spatial patterns of the prokaryotic plankton. Moreover, multiple Labyrinthulomycetes ecotypes with distinct vertical patterns were detected and, based on the physiologic, metabolic, and genomic understanding of their cultivated relatives, were inferred to play multifaceted key roles in the carbon cycle and sequestration, particularly as contributors to the vertical carbon export from the surface to the dark ocean, i.e., the biological pump.

## INTRODUCTION

Heterotrophic microbial plankton play a fundamental role in secondary production and driving marine carbon cycles in the world’s oceans (1–3). Their communities are shaped by environmental factors and resources and also have profound influences on marine ecosystem function and global climate change (4–7). Although tremendous efforts have been made to explore community patterns, environmental drivers, and ecological functions of marine prokaryotic heterotrophs, those of their microeukaryotic counterparts remain largely unknown (8, 9). Particularly, heterotrophic protists, which have increasingly been reported to be a dominant microbial component of deep-sea sediments and pelagic waters, lack sufficient data on their diversity and ecological roles (10–12).

Labyrinthulomycetes are an important protistan group of mainly osmo-heterotrophic microeukaryotes, and their biomass can surpass that of prokaryotic plankton in coastal oceans, largely due to their large cell volumes and carbon contents (i.e., over 1,000 times that of prokaryotic cells) (13–16). Like many other marine microorganisms, this class of heterotrophic microeukaryotes shows partitioning by seasons and/or coastal habitats (e.g., estuarine, nearshore, and offshore waters) (9, 15). Interestingly, in spite of a few dominant and prevalent phylotypes, most phylotypes of this class exhibit transient blooms in coastal waters, which are distinct from the patterns of the prokaryotic plankton (9). While relatively few studies have examined the Labyrinthulomycetes communities and functions in the pelagic oceans, several lines of evidence indicate their widespread distributions and significant contribution to the deep-sea (e.g., marine snow, sediments, and bathypelagic waters) biomes (10, 12, 17). Our recent investigation in the South China Sea provided the first report of their vertical community structure in pelagic waters and revealed their potential as a key player in both dark ocean carbon sequestration and remineralization processes through stratification of different ecotypes (12). Beyond most strains’ abilities to secrete a range of extracellular hydrolases to degrade both terrestrial and phytoplankton-derived detritus (18), Labyrinthulomycetes protists have the potential to facilitate aggregation of organic particles through their ectoplasmic nets and exopolysaccharides (19, 20) and thus are considered to promote the vertical export of organic particles from the surface to deep oceans for long-term carbon storage (i.e., the biological pump) (12). However, the community dynamics and ecological roles of the Labyrinthulomycetes protists in the pelagic oceans clearly deserve study across broader ocean realms. Particularly, spatial variation in their biomass, diversity, and community composition in pelagic waters, especially in the horizontal scale, has not been well described. Here, we hypothesize that pelagic Labyrinthulomycetes exhibit patchy distributions and niche partitioning similar to those observed in coastal waters (9, 13, 15) and, therefore, can impose distinct and multifaceted influences on the oceanic carbon cycling and sequestration.

**FIG 1:**
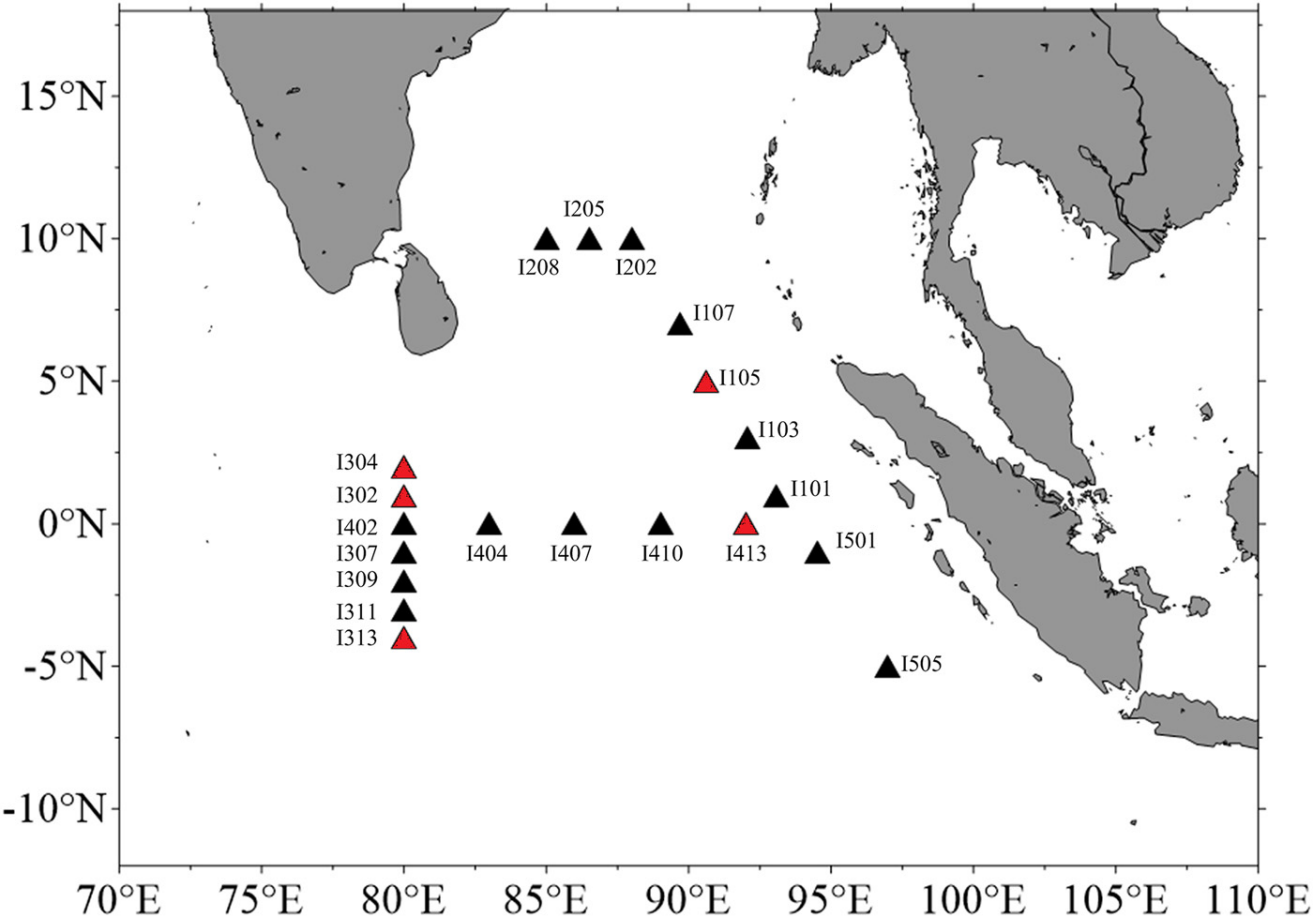
Sampling stations of the Eastern Indian Ocean cruise during intermonsoon 2018. All the stations except I501 and I505 were sampled for flow-cytometric analysis, and those marked with red were found to be the Labyrinthulomycetes-bloom stations (as defined in Fig. 2). Stations I105, I107, I202, I205, I208, I302, I309, I313, I402, I407, I413, I501, and I505 were selected for sequencing of the Labyrinthulomycetes 18S rRNA gene.

To test these hypotheses, we combined flow-cytometric and molecular tools to observe spatial dynamics of Labyrinthulomycetes communities from epipelagic to bathypelagic waters across multiple transects in the Eastern Indian Ocean, which covers the equator, Bay of Bengal, and western offshore area of Indonesia (Fig. 1). Cell abundance and biomass of prokaryotic plankton were also determined as a comparative reference. The ultimate goal of this study is to better understand the community patterns, potential niche partitioning, and ecological implications of this historically overlooked nutrient-remineralizing protistan group in the pelagic ocean, increasing our knowledge in the ecology of marine heterotrophic microeukaryotes.

## RESULTS AND DISCUSSION

### Abundance pattern and biomass contribution of Labyrinthulomycetes.

Seawater samples for flow cytometry enumeration of Labyrinthulomycetes protists and prokaryotic plankton were collected from depths between 5 and 2,000 m at 18 stations in the Eastern Indian Ocean (Fig. 1). The average and maximum abundances of the total Labyrinthulomycetes protists were 359 and 2,640 cells mL^−1^, equivalent to 7.4 and 54.4 μg C L^−1^ of biomass, respectively. In comparison, the average and maximum biomasses of the prokaryotic plankton were estimated to be 5.7 and 45.5 μg C L^−1^, respectively, slightly lower than those of the Labyrinthulomycetes. The overall higher biomass of Labyrinthulomycetes compared to that of the prokaryotic plankton was also observed in two of the three previous cruises in the Equatorial Indian Ocean, although it was during a different intermonsoon period (September to October) from that of our study (March to April) (21). Here, our results over a larger spatial scale reinforced the significant contribution of the Labyrinthulomycetes protists to the living carbon stock of this pelagic ocean realm.

**FIG 2:**
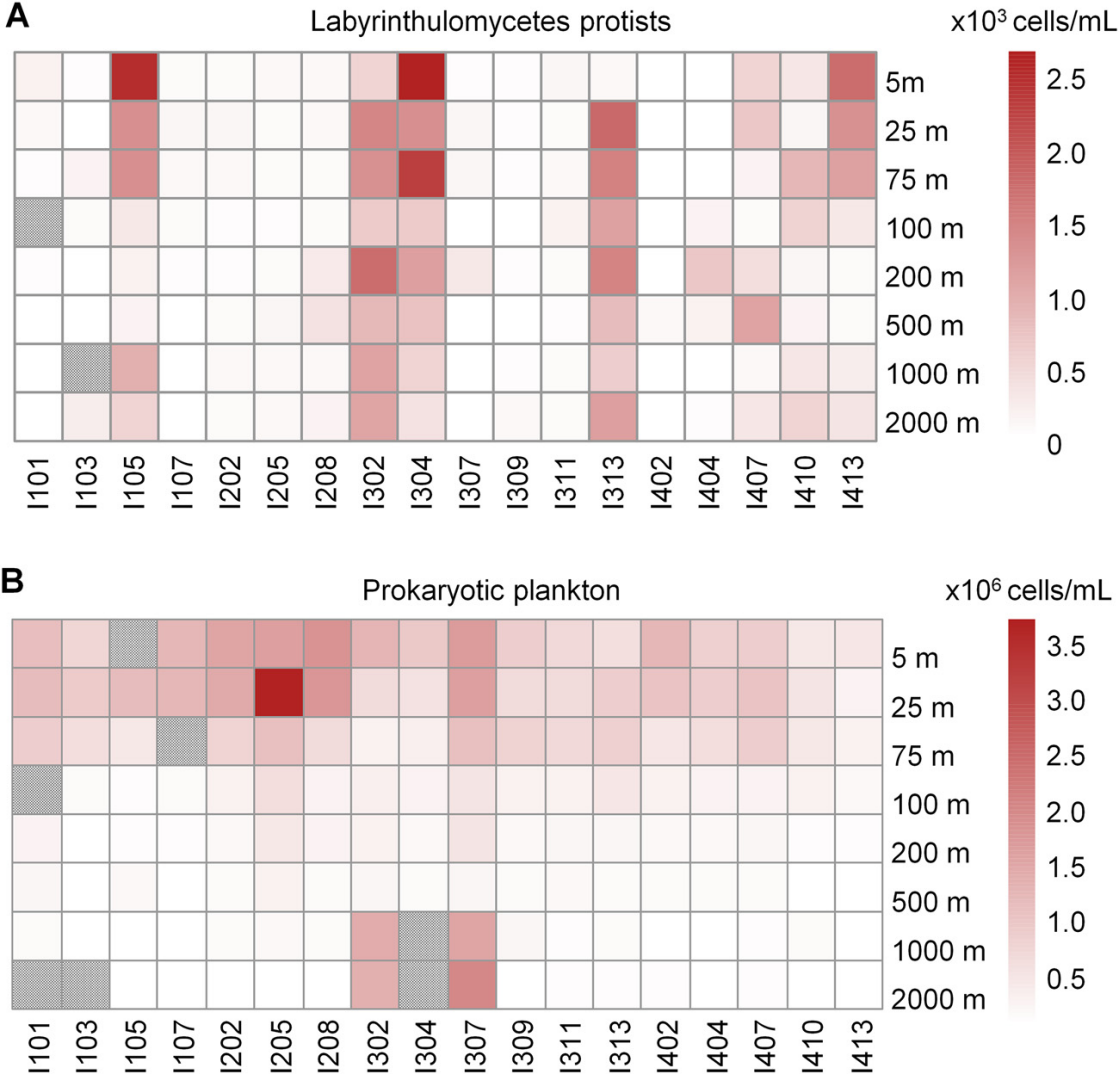
Spatial patterns in cell abundances of Labyrinthulomycetes protists (A) and prokaryotic plankton (B) across different stations and depths in the pelagic waters of the Eastern Indian Ocean. Stations I105, I302, I304, I313, and I413 exhibited >1,000 cells mL^−1^ of Labyrinthulomycetes at the surface layer (5 m or 25 m) and were defined as Labyrinthulomycetes-bloom stations. Gray shading represents samples that were not analyzed for cell abundances.

While prokaryotic abundance rapidly declined with depth, the Labyrinthulomycetes abundance was relatively constant throughout the water column within a given station (Fig. 2). This finding is consistent with recent observations in the pelagic South China Sea, where the biomass of Labyrinthulomycetes was found to approach and even exceed that of prokaryotic plankton in the mesopelagic and bathypelagic waters, respectively (12). Together with the previous reports on the major contribution of Labyrinthulomycetes 18S rRNA sequences and biomass to the heterotrophic communities of the deep-sea sediments and marine snow (10, 17), we consider this heterotrophic group of microeukaryotes to be an important and active component in the dark ocean carbon cycling and sequestration.

Interestingly, the horizontal distribution of the Labyrinthulomycetes was much patchier than that of the prokaryotic plankton, and stations with Labyrinthulomycetes blooms at the surface (>1,000 cells mL^−1^ at 5 or 25 m) generally had persistently high abundance at depth (Fig. 2A), with the average abundance over all depths of these “bloom” stations (I105, I302, I304, I313, I413) ranging from 642 to 1,202 cells mL^−1^, several times higher than that of the other stations (analysis of variance [ANOVA], *P* < 10^−7^). ANOVA also indicated that the abundance of Labyrinthulomycetes cells differed significantly among stations (*P* < 10^−15^) but not significantly among depths (*P* > 0.05). In the pelagic waters of the South China Sea, the Labyrinthulomycetes abundance was also reported to show a minimal vertical change, but blooms at certain stations were not observed (12). Here, our larger-scale investigation in the pelagic waters of the Eastern Indian Ocean provided the first report of the patchy horizontal distribution of the Labyrinthulomycetes in pelagic waters, which is distinct from the pattern of the prokaryotic plankton (Fig. 2B) but is consistent with the short-term bloom dynamics of these protists at the coastal time series in Japan, Northern China, and North Carolina (USA) (9, 13, 15). Considering the larger cell size and sinking capability of Labyrinthulomycetes (12, 22), we speculate that these distribution patterns were caused by their patchy blooms in the upper ocean and sinking, making this a potentially understudied and spatially variable contribution to the biological pump. Thus, it is not surprising to find that unlike the prokaryotic abundance, the Labyrinthulomycetes abundance was difficult to predict by continuous oceanographic gradients (Table S1). In our observations, prokaryotic abundance showed significant correlations (*P* < 0.01) with most of the measured, generally depth-associated environmental parameters, including temperature, salinity, dissolved oxygen, pH, silicate, phosphate, nitrate, and total phosphate, but the Labyrinthulomycetes abundance showed only weak correlations (*R* ≈ 0.2, *P* < 0.05) with few environmental parameters (nitrite, total phosphorous, temperature, and dissolved oxygen). Additionally, most of the stations with high Labyrinthulomycetes abundance were located in the equatorial region, where the prokaryotic abundance was relatively low. However, the Bay of Bengal, which was at a higher latitude and was an acute oxygen minimum zone (23), harbored abundant prokaryotic plankton but fewer Labyrinthulomycetes protists. As has been reported in coastal waters (9), these results suggest potentially distinct ecological niches, strategies, and roles of the Labyrinthulomycetes protists compared to those of the prokaryotic plankton in the pelagic ocean.

Although many Labyrinthulomycetes are presumed to sink, their cell abundance is also dependent on processes occurring throughout the water column, including bottom-up controls by resources and environmental conditions as well as top-town controls by zooplankton predation and virus lysis (9, 12, 24–26). Their persistently high biomass throughout the whole water column at some stations (e.g., I302, I304, and I313; Fig. 2A) could be attributed to both their sinking potential and their robustness in deep-sea environments (e.g., ability to use recalcitrant organic matter at low temperature; resistance to predation/lysis). However, the differences among stations raise the question of why the Labyrinthulomycetes blooms occurred at specific stations. As the Labyrinthulomycetes protists were often observed to feed on or live with algae-derived particles (27, 28), we had predicted that Labyrinthulomycetes blooms in the upper ocean could be triggered by phytoplankton blooms; however, chlorophyll concentration did not significantly differ at stations with Labyrinthulomycetes blooms (ANOVA, *P* > 0.05; Fig. S1). In addition, the Labyrinthulomycetes cell abundance in the epipelagic zone was not related to chlorophyll or any other environmental parameters (*P* > 0.05). Thus, we hypothesized that the Labyrinthulomycetes blooms could be driven by specific populations or ecotypes, which in turn suggests the need to characterize the Labyrinthulomycetes at finer taxonomic resolution for a deeper insight into their ecological distribution and roles in oceanic carbon cycling and sequestration.

Overall, taking the prokaryotic plankton as a reference, our observations in the pelagic waters of the Eastern Indian Ocean clearly revealed a distinct spatial abundance pattern of the Labyrinthulomycetes protists as well as their greater relative importance in the dark ocean carbon cycling and sequestration.

### Spatial patterns in Labyrinthulomycetes diversity and composition.

In order to further understand the diversity patterns and community-level distributions of the Labyrinthulomycetes protists in the pelagic Eastern Indian Ocean, representative samples from different depths of stations I105, I107, I202, I205, I208, I302, I309, I313, I402, I407, I413, I501, and I505, which covered different regions and contained both the Labyrinthulomycetes bloom and nonbloom stations (Fig. 1), were subjected to high-throughput sequencing of the Labyrinthulomycetes 18S rRNA gene. The Deblur workflow resolved 2,167 amplicon sequence variants (ASVs) from 93 samples, and 723 ASVs were annotated to the class Labyrinthulomycetes using the expert-curated PR2 database (29). These ASVs were cross-verified to be Labyrinthulomycetes using the more inclusive SILVA SSU database (30), which provided poor classification at lower taxonomic levels but predicted additional ~500 ASVs to be Labyrinthulomycetes. As the main goal of this study is to exam community patterns rather than to find novel species, we used the more conservative and specific PR2 annotations. After rarefaction, 676 ASVs and 89 samples were retained to compare the diversity and community composition of Labyrinthulomycetes. This data set included both known genera, i.e., *Aplanochytrium*, *Aurantiochytrium*, *Oblongichytrium*, *Thraustochytrium*, *Schizochytrium*, and *Ulkenia*, as well as many unclassified taxa within the families Labyrinthulaceae and Thraustochytriaceae, or within the class Labyrinthulomycetes (Table S2), suggesting a high diversity of the Labyrinthulomycetes protists in the pelagic waters of the Eastern Indian Ocean.

**FIG 3:**
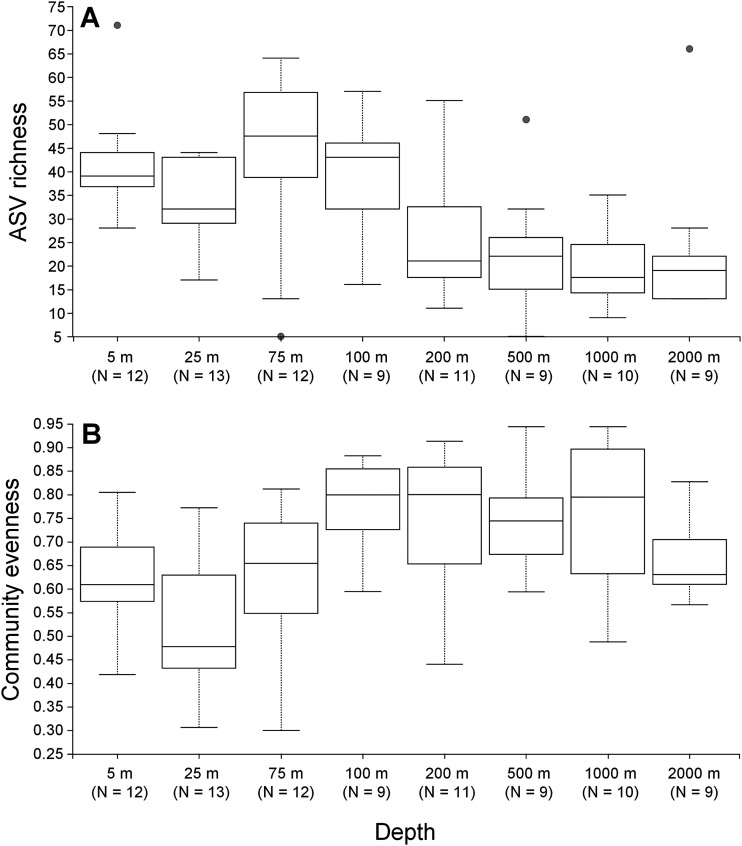
Vertical variations in ASV richness (A) and Pielou’s community evenness (B) of the Labyrinthulomycetes protists in the pelagic waters of the Eastern Indian Ocean. Groups with small sample size (50 m and 300 m, N < 3) are excluded from the boxplots.

Surprisingly, most ASVs were observed in one to few samples, suggesting specialized habitats. The observed ASV richness of individual samples (Fig. 3A) ranged from 5 to 71, with a significant difference between both water depths (*P* = 0.0001) and stations (*P* = 0.0233). Richness was generally higher in the epipelagic than in the dark ocean, with a peak around the deep chlorophyll maximum layer (75 m). Stations I105, I302, I131, and I402, which exhibited Labyrinthulomycetes blooms, showed slightly but not consistently lower richness than the nonbloom stations (*P* = 0.0468) (Fig. S2). The community evenness (Fig. 3B), however, showed a significant difference with depth (*P* = 0.0020) but not stations (*P* = 0.4165); unlike richness, the evenness increased at depths of ≥100 m. The contrasting vertical profiles between richness and evenness (Fig. 3) resulted in less significant difference in Shannon’s diversity across depths (*P* = 0.0436), with the greatest values at the bottom of the euphotic layer (200 m) (Fig. S3A). Additionally, Shannon’s diversity showed significant difference among stations (*P* = 0.0440) and was generally higher in the Bay of Bengal (stations I107, I202, I205, I208) as well as in the cross-equatorial transect (stations I302, I309, I313, I402) (Fig. S3B). Unlike Labyrinthulomycetes cell abundance, ASV richness and community evenness were closely related to largely depth-related environmental parameters while Shannon’s diversity was not linearly correlated with any of the measured environmental factors (Table S3). Overall, these results revealed predictable vertical diversity profiles of the Labyrinthulomycetes protists; although their diversity also varied among stations, the difference between the bloom and nonbloom stations was not apparent.

**FIG 4:**
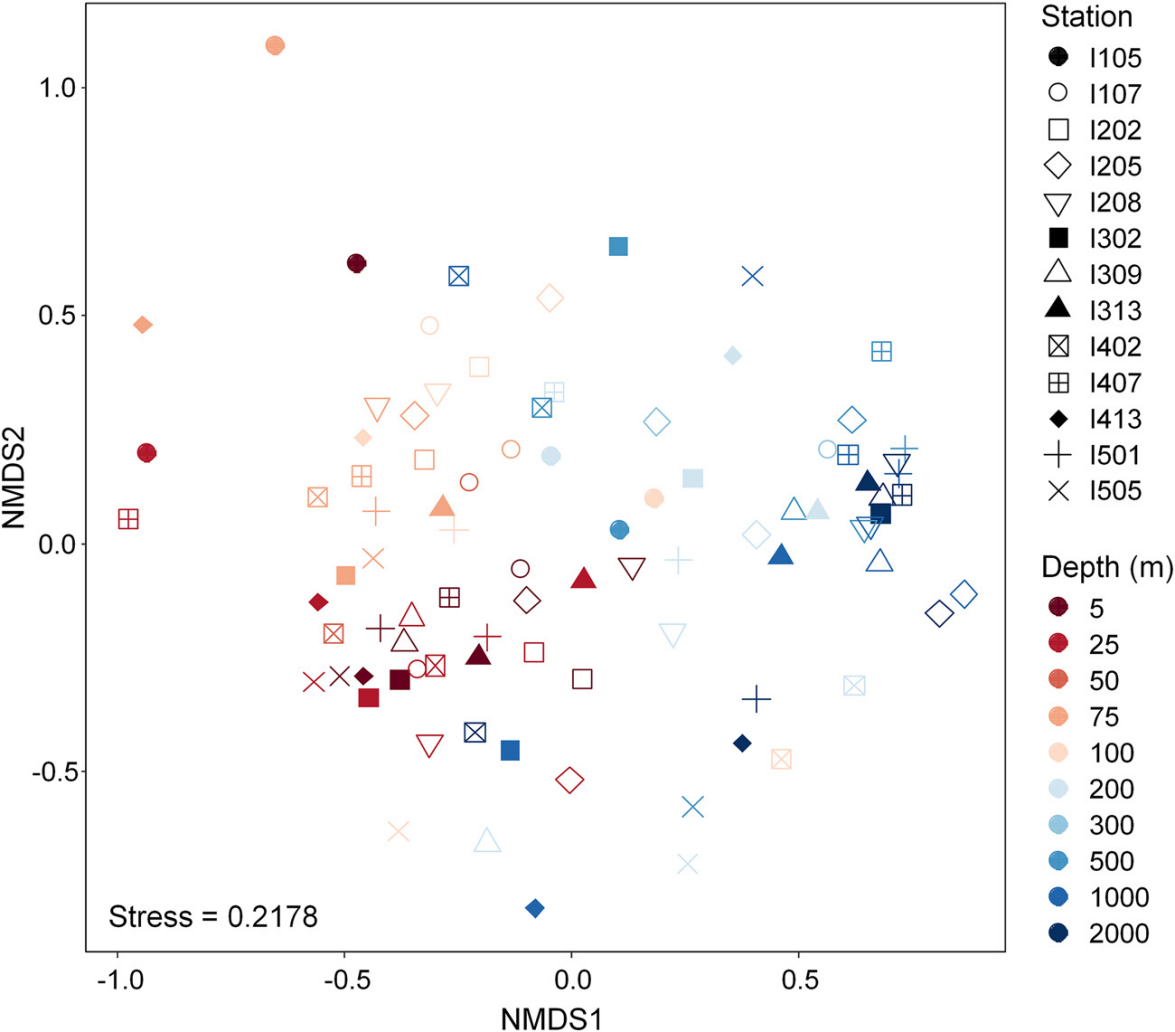
NMDS ordination based on the Bray-Curtis dissimilarity matrix showing the spatial variation of Labyrinthulomycetes community composition at the ASV level. Shapes with solid color fills represent stations with Labyrinthulomycetes blooms as defined in Fig. 2.

To reveal the spatial community structure of the Labyrinthulomycetes, we further examined ASV composition among stations and depths after confirming equal dispersal (Betadisper, *P* > 0.05). Adonis tests based on the Jaccard distance matrix (presence or absence) and the Bray-Curtis dissimilarity matrix (relative abundance weighted) both suggested significant patterns vertically (*P* = 0.001) and across stations (*P* = 0.035 for Jaccard and *P* = 0.029 for Bray-Curtis), although the difference was not significant between bloom and nonbloom stations (*P* > 0.05). In contrast, the prokaryotic plankton in the same region exhibited high similarity among different stations at the same depth (31). However, these data as well as nonmetric multidimensional scaling (NMDS) ordination based on Bray-Curtis distance (Fig. 4) suggested an influence of sample depth greater than that of station on Labyrinthulomycetes composition. Generally, adjacent water layers harbored more similar communities, while greater dissimilarities existed between the surface and deep ocean. Pairwise comparisons by permutational multivariate ANOVA (PERMANOVA) tests provided statistical details (Table S4): communities at different depths showed no significant difference (*P* > 0.05) either within the upper epipelagic (≤50 m) or within the aphotic zone (≥200 m); significant dissimilarities existed mostly between the distant water layers (*P* < 0.05). However, it is worth mentioning that the statistical results that communities at 50 m were not different from communities at any other layers (*P* > 0.05) and that communities at 300 m differed only from communities at 5 m can be attributed to the small sample size (*n* = 2) of 50 m and 300 m.

**FIG 5:**
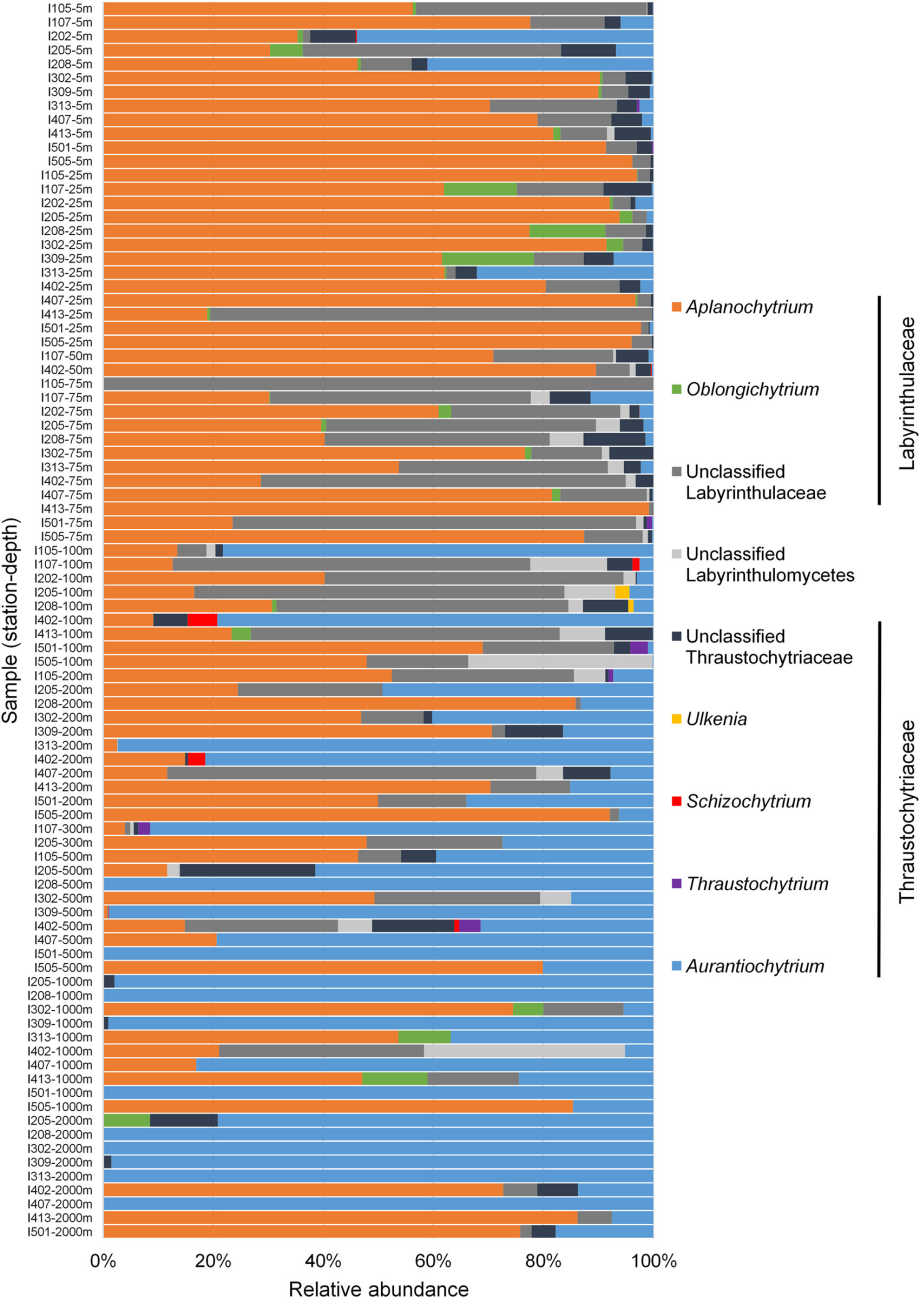
Vertical variations in Labyrinthulomycetes composition at genus and family levels.

Vertical difference in community composition was also apparent at the genus level, with *Aplanochytrium* and *Aurantiochytrium* generally dominant in the epipelagic and deep-sea communities, respectively (Fig. 5). The genus *Aplanochytrium* has been reported to be a major component of Labyrinthulomycetes in the pelagic waters of South China Sea (12) and at the coast (9, 13). The cultured *Aplanochytrium* strains exhibit multiple trophic strategies (e.g., feeding on dead or living algae) and can potentially facilitate the formation of large and fast-sinking aggregates through ectoplasmic nets and exopolysaccharides (28, 32). They are also reported to be associated with phytoplankton and zooplankton in the epipelagic ocean (24), suggesting their important influence on planktonic food webs. In our observations, this genus was still abundant in many of the deep-sea samples, which may be attributed to *in situ* growth or export from the epipelagic layer. Therefore, as the dominant genus, *Aplanochytrium* is likely to accelerate the biological pump. Unlike *Aplanochytrium*, the *Aurantiochytrium* are typical saprophytes (32) and well documented to produce large amounts of fatty acids (33), with some strains observed to graze on bacteria and other organic particles at their ameboid phase (34), and thus might play a key role in decomposition and conversion of organic matter. Members of this genus are often observed in decaying mangrove leaves and adjacent seawaters and sediments (35–37), but both experimental evidence and genomic evidence suggest relatively poor cellulase activity in the *Aurantiochytrium* strains (32, 38), indicating that they likely cometabolize the plant-derived organic matter with other heterotrophs (e.g., fungi). Recently, this genus has also been reported to be abundant in the bathypelagic waters of the South China Sea (12). In the present study, we observed even greater dominance of *Aurantiochytrium* in the dark ocean, with several samples consisting of exclusively *Aurantiochytrium* ASVs (Fig. 5). Their production of extracellular hydrolases and intracellular fatty acids in deep-sea environments are still in need of future confirmation, but our results indicate their potential importance in regulation of deep-sea food webs and carbon sinks through their distinct trophic modes and efficient secondary production. Particularly, they could play roles as major components of the “lipid pump” (39) or as valuable dietary components for the zooplankton in the dark ocean.

To explore potential impact of environmental factors on the Labyrinthulomycetes communities, we analyzed the correlations between the Labyrinthulomycetes composition and a range of environmental parameters that contained no missing values, including temperature, salinity, dissolved oxygen, pH, density, and chlorophyll, in terms of conditional effects. Stepwise selection for the redundancy analysis (RDA) model identified temperature, salinity, dissolved oxygen, and chlorophyll as significant factors for the Labyrinthulomycetes composition at the ASV level (Table S5) and temperature, density, salinity, and depth to be significant at the genus level (Table S6). Given that these parameters are typically related to depth (Spearman correlation tests, *P* < 0.001; Table S7), the RDA results reinforced the vertical partitioning of the Labyrinthulomycetes communities. Unlike the monotonic changes in temperature, salinity, and density with depth, however, the oxygen minimum and chlorophyll maximum are usually in 200 to 1,000 m and 50 to 100 m, respectively. Therefore, compared to that at the genus level, higher taxonomic resolution at the ASV level suggested potentially more complex drivers for the Labyrinthulomycetes communities.

In spite of differences in abundance across locations, our analysis revealed obvious vertical but minor horizontal changes in the diversity and taxonomic composition of the Labyrinthulomycetes communities in the pelagic waters of the Eastern Indian Ocean, with the genera *Aplanochytrium* and *Aurantiochytrium* particularly inferred to be important heterotrophic protistan players in the carbon cycle and storage processes of the epipelagic and deep-sea ecosystems, respectively.

### Depth-associated ecotypes of Labyrinthulomycetes.

**FIG 6:**
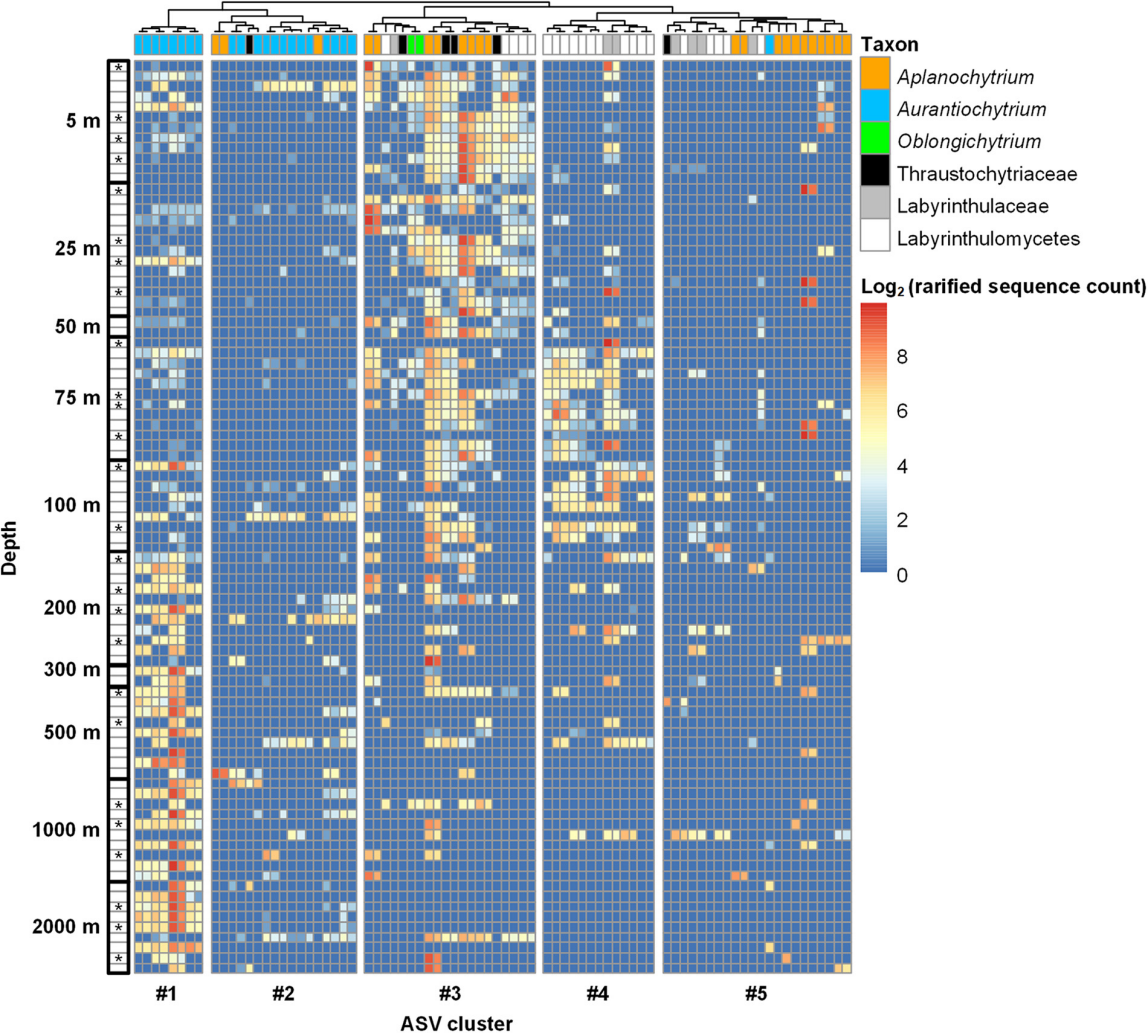
Heatmap showing the vertical distribution patterns of the 80 most abundant Labyrinthulomycetes ASVs whose relative abundance was higher than 0.1% in the total samples. Samples (rows) were sorted by depth, and those marked with asterisks were from the Labyrinthulomycetes-bloom stations as defined in Fig. 2. ASVs (columns) were annotated with their taxonomic groups at the genus or the most specific classified level. Ward’s hierarchical clustering was employed to group the ASVs into potential ecotypes with differential distribution patterns.

In order to gain deeper insights into the community structure as well as potential niche partitioning of the Labyrinthulomycetes, we extracted the 80 most abundant ASVs whose average relative abundance across all samples was greater than 0.1% (totally 89.3%) (Fig. 6). Hierarchical clustering resolved these ASVs into 4 ecotypes (in 5 clusters) that exhibited distinct vertical distribution patterns (Fig. 6). Based on metabolic information from cultured strains, we also evaluated the potential roles of these different ecotypes in pelagic carbon cycling and storage.

The first ecotype (cluster 1, Fig. 6) comprised 8 ASVs, all identified as *Aurantiochytrium*, that occurred throughout the water column but were more abundant and prevalent below 200 m than above. The enrichment of these ASVs in the deep ocean could indicate their potential utilization and conversion of recalcitrant deep-sea organic matter or reflect the accumulation of cells from lower-density epipelagic waters (12). Previous studies reported significant presence of Labyrinthulomycetes in deep-sea marine snow and sediment (10, 17), but their taxonomic composition and functions were not resolved. The present research, aligning with our recent observations in pelagic waters of the South China Sea (12), revealed members of *Aurantiochytrium* as a major component of the Labyrinthulomycetes communities in the deep waters. Given typically high accumulation of fatty acids in *Aurantiochytrium* cells (33), this ecotype could play an important role in the deep-sea food webs and long-term carbon storage. In future, sinking particles should be separately sampled and analyzed for the carbon metabolic functions of different Labyrinthulomycetes taxa to test this hypothesis.

The second ecotype (clusters 2 and 5, Fig. 6) comprised the majority of the ASVs (including the rare ASVs not shown). They occurred sparsely at different depths but reached high relative abundance in some samples. This ecotype consisted of members that belonged to *Aplanochytrium*, *Aurantiochytrium*, and other taxonomic groups. It is also worthwhile to note that ASVs within the same genus of this ecotype often cooccurred. This trait separates the two clusters, but we consider them as one ecotype because of their shared spatially patchy distribution pattern. Similar to the short blooms and patchy distributions in the coastal waters of North Carolina (9), the distribution pattern of this ecotype could be attributed to specific microhabitats, hosts, or substrates that were sparse and patchy in the ocean or strong top-down control by zooplankton predation or virus lysis. This population pattern is rare in marine prokaryotic communities (40); thus, the driver behind these patterns represents an area of active investigation.

The third ecotype (cluster 3, Fig. 6) comprised 8 *Aplanochytrium* ASVs, 2 *Oblongichytrium* ASVs, and several unclassified ASVs. This ecotype was most prevalent and abundant in the epipelagic zone, becoming relatively rare or patchy below about 100 m depth. The genus *Aplanochytrium* has been reported to interact with zooplankton and phytoplankton via diverse trophic activities, including parasitism, symbiosis, saprotrophy, and predation (24, 28, 32, 41, 42). Its extensive distribution in the epipelagic ocean suggests its potential importance in the grazing and detrital food chains. Some members of this genus have recently been found to graze on living diatoms by ectoplasmic nets and support the formation of large, fast-sinking particles by secretion of extracellular polysaccharides (28), and thus, they can potentially drive the vertical carbon export as a novel player in the biological pump (12). Our results showed persistent occurrence of some *Aplanochytrium* ASVs across different water layers from the surface until the bathypelagic layer, providing a preliminary line of *in situ* evidence for their vertical export, but future investigation is still needed to quantitively evaluate their effect on ocean carbon fluxes.

The fourth ecotype (cluster 4, Fig. 6) was enriched around the deep chlorophyll maximum (50 to 100 m) but rare in both the surface and the deep ocean. This distinct distribution pattern could be attributed to potential association to the phytoplankton or phytoplankton-derived fresh organic matter, in which case, this ecotype could play a key role in carbon conversion and trophic transfer in this layer. However, members of this ecotype were mostly unclassified Labyrinthulomycetes, and we therefore lack metabolic information from closely related strains to aid in functional predictions of the carbon cycle. To gain insights about these uncultured and unclassified taxa, novel *in situ* function-targeted approaches (e.g., metagenomics/transcriptomics) should be developed and employed in future studies.

Overall, the differential vertical distribution patterns of the dominant Labyrinthulomycetes ASVs in the pelagic waters of the Eastern Indian Ocean suggested that they exhibit niche partitioning and potentially play multifaceted roles in pelagic carbon cycling and storage processes. The results were generally consistent with our previous findings in the pelagic waters of the South China Sea, except that we did not find an Eastern Indian Ocean ecotype that was abundant in both the surface and bathypelagic layers but rare in the middle layers (12). In our South China Sea study, this ecotype was interpreted to comprise K-selected specialists that prevail in the surface environment with intense competition and in the bathypelagic environment with poor resource availability, with low abundance in middle layers reflecting heavy grazing pressure by the deep-euphotic and mesopelagic zooplankton or rapid sinking through the middle layers with accumulation in the high-density bathypelagic waters (12). The absence of this ecotype in the Eastern Indian Ocean could indicate differential ecological pressures shaping the Labyrinthulomycetes communities in different pelagic ocean realms.

### Key contributing members of the patchy Labyrinthulomycetes blooms.

**FIG 7:**
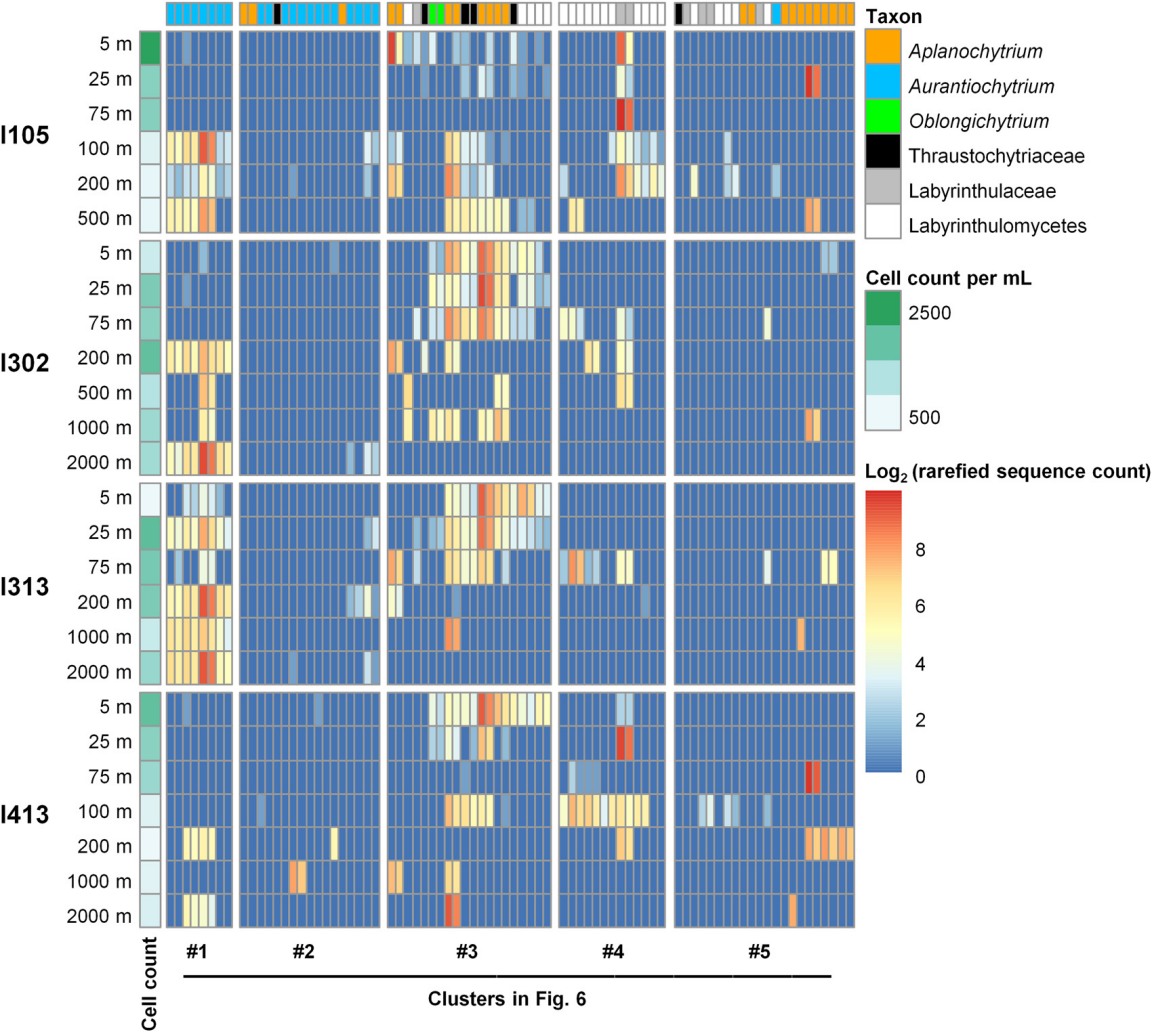
Heatmap showing the distributions of the 80 abundant Labyrinthulomycetes ASVs (whose relative abundance was higher than 0.1% in the total samples) at the Labyrinthulomycetes-bloom stations as defined in Fig. 2. Samples (rows) were grouped by stations and then ordered by depth for each station and were annotated with the total Labyrinthulomycetes cell abundance. ASVs (columns) were arranged by the consistent order of Fig. 6 and were annotated with their taxonomic groups at the genus or the most specific classified level.

While no significant difference in Labyrinthulomycetes diversity and composition was identified between the bloom and nonbloom stations, we hypothesize that some prevalent taxa could make a major contribution to the patchy Labyrinthulomycetes blooms. To discover whether the high abundance of total Labyrinthulomycetes cells at specific stations was driven by certain key contributing ASVs, we rearranged the heatmap of the 80 most abundant ASVs (whose average relative abundance across all samples was greater than 0.1% and in total accounted for 89.3% of sequences) by illustrating their vertical distributions at each of the bloom stations, including I105, I302, I313, and I413 (Fig. 7). While the total Labyrinthulomycetes cells were also abundant at I304, samples of this station were not sequenced and therefore not included in the heatmap. The bloom stations subjected for sequencing were geographically distant from each other (Fig. 1). As a result, several prevalent ASVs, belonging mostly to clusters 1 and 3 in Fig. 6, were found consistently to be major components of the Labyrinthulomycetes communities at these bloom stations, though their relative abundance varied among depths and stations (Fig. 7). Since their relative abundances were also high in the nonbloom stations (Fig. 6 and Fig. S4), these key ASVs might have a specific niche that allows them to reach high abundances only under certain conditions occurring at these bloom stations but remain to be important components under other conditions at all the other stations.

Generally, these key contributing members in clusters 1 and 3 showed contrasting preferences to the deeper and upper layers, respectively (Fig. 6 and 7 and Fig. S4). However, at station I105, ASVs of cluster 3 exhibited relative abundance in the deeper layers exceptionally higher than that in the upper layers (Fig. 7). Clearly, the high Labyrinthulomycetes abundance at the upper epipelagic zone (5 to 75 m) of I105 was largely contributed by a few ASVs that were less prevalent but occasionally dominant, rather than by those typical major components of the upper epipelagic communities at the other bloom stations. It is also worthwhile to note that many of the 80 abundant ASVs in the total samples, particularly members of clusters 2 and 5, were absent or only occasionally present at these bloom stations (Fig. 7), suggesting limited habitats of these ASVs in the horizontal scale. Generally, these patchily distributed ASVs can also play a role in the station-specific blooms of Labyrinthulomycetes protists but appear to contribute less than the prevalent ASVs.

### Conclusions.

Over the past few decades, the important role of Labyrinthulomycetes in coastal marine ecosystems has been well documented, but their diversity and distribution patterns in the pelagic ocean remain poorly understood (9, 12, 13). This study revealed a patchy distribution of Labyrinthulomycetes in the pelagic waters of the Eastern Indian Ocean, perhaps indicating ephemeral blooms linked to persistently high biomass throughout the water column that suggest potential sinking and vertical carbon export to the deep ocean. Remarkably, the average biomass of Labyrinthulomycetes exceeded that of the prokaryotic plankton, especially in the bathypelagic waters, suggesting their significant contribution to the particulate carbon stock and storage of the pelagic ocean. High-throughput sequencing further revealed high diversity of Labyrinthulomycetes at the ASV and genus levels, with richness decreasing while evenness increased with depth and Shannon’s diversity peaked in the lower epipelagic layer (100 to 200 m). The community composition of Labyrinthulomycetes showed significant variations across both vertical and horizontal scales but was more associated with depth. Particularly, *Aplanochytrium* dominated the Labyrinthulomycetes communities in the epipelagic ocean while *Aurantiochytrium* was dominant in the dark ocean. However, most ASVs were detected across multiple water column layers, suggesting their strong environmental adaptability and dispersal (sinking) abilities. Hierarchical clustering resolved the abundant ASVs (>0.1% in total samples) into ecotypes with distinct vertical patterns, suggesting their differential niches and multifaceted potential roles in the carbon cycling and biological pump of the pelagic ocean. In particular, the majority of ASVs showed highly patchy distributions as previously found in the coastal waters of North Carolina (9), reinforcing the distinct strategies, drivers, and functions of this protistan heterotrophic group compared to those of their prokaryotic counterparts. In spite of variabilities across stations in Labyrinthulomycetes diversity and composition, the differences were not apparent between the stations with high and low abundance of total Labyrinthulomycetes cells, but some prevalent ASVs are found to make a major contribution to the patchy Labyrinthulomycetes blooms, leaving their specific triggers and driving processes in need of future studies.

## MATERIALS AND METHODS

### Seawater sampling and environmental characterization.

Seawater samples were collected using the Sea Bird CTD rosette sampler from the epipelagic (5 m, 25 m, 50 m, 75 m, 100 m), mesopelagic (200 m, 300 m, 500 m), and bathypelagic (1,000 m, 2,000 m) layers during the intermonsoon period (March 25 to April 30) of 2018 in the Eastern Indian Ocean (Fig. 1). Temperature, salinity, dissolved oxygen, pH, and chlorophyll were detected by the conductivity-temperature-depth (CTD) sensors, and the nutrients were analyzed in lab as described previously (43, 44). Triplicate subsamples of 4 mL seawater were fixed with 0.22-μm-filtered glutaraldehyde (0.5% final concentration) and stored at −80°C for flow-cytometric enumeration of Labyrinthulomycetes and prokaryotic plankton (12, 44). An additional 3 L of seawater was filtered through 0.22-μm polycarbonate Isopore membrane filters (Millipore, USA), and the resulting filters were stored at −80°C until DNA extraction.

### Biomass estimation of Labyrinthulomycetes and prokaryotic plankton.

The cell abundances of Labyrinthulomycetes and prokaryotic plankton were determined by a fluorescence-activated cell sorter (FACS) Calibur flow cytometer (BD-Biosciences, USA) following the previously described protocols (12, 13, 45), using the Labyrinthulomycetes-specific dual fluorescent dye acriflavine-HCl (Sigma, Germany) and the DNA dye SYBR-I green (Molecular Probes, USA), respectively. Green-fluorescent polystyrene latex beads (Molecular Probes, USA) were used as an internal standard to calculate the cell counts. To keep consistency and comparability with the previous studies (12, 21), the average cellular carbon contents of 2.06 × 10^−11^ g C (22) and 1.24 × 10^−14^ g C (46) were used for estimating the biomass of Labyrinthulomycetes and prokaryotic plankton in the oceanic water samples, respectively.

### Labyrinthulomycetes 18S rRNA gene sequencing.

The total DNA of individual samples was extracted using E.Z.N.A. water DNA kit (OMEGA, USA). The 18S rRNA gene of Labyrinthulomycetes was amplified using the barcodes-linked primers LABY-A (5′-GGGATCGAAGATGATTAG-3′) and LABY-Y (5′-CWCRAACTTCCTTCCGGT-3′), as described previously (9, 12, 13). High‐throughput sequencing of the purified and pooled PCR products was performed on an Illumina HiSeq 2500 platform at Biomarker Technologies Co, Ltd., Beijing, China.

### Bioinformatic analyses.

Raw sequences were demultiplexed and assigned to corresponding samples using Illumina CASAVA software. The pair-end sequences were preprocessed using the fastp software (47). Primer nucleotides were first removed from the front of both forward and reverse reads, and poor-quality nucleotide tails with a quality score (Q) of <30 were also dropped in order to yield higher merging rates. Then, the trimmed paired-end sequences were merged when they had a ≥10 bp overlap with ≤3 mismatches, as described previously (9). Next, the merged sequences were imported into the QIIME 2 platform (48) and subject to basic quality score-based filtering (i.e., default settings of the plugin “quality-filter”), as recommended, prior to performing the Deblur workflow. With the sequences truncated at the length of 390 bp and the positive filtering database specified to be Protist Ribosomal Reference database (PR2 version 4.13.0) (29), the Deblur software plugin (49) further denoised the sequences and retained amplicon sequence variants (ASVs) that appeared ≥10 times across all samples. The resulting ASVs were annotated by the BLAST+ consensus taxonomy classifier (50) against the PR2 database, and those not annotated to the class of Labyrinthulomycetes were discarded. After examination of rarefaction curves, the filtered sequences were finally rarified to an even depth of 1,401 sequences per sample for downstream statistical analyses and visualization.

### Statistical analyses.

The alpha (ASV richness, Pielou’s evenness, and Shannon’s diversity) and beta (Jaccard and Bray-Curtis distance matrixes) diversity metrics were calculated by the QIIME 2 “core-metrics” pipeline, and their diversity patterns were compared using Kruskal-Wallis, Adonis, and PERMANOVA tests. The spatial patterns in Labyrinthulomycetes composition at the ASV level were visualized by nonmetric multidimensional scaling (NMDS) ordination using the R package “*vegan*” (51). The relationship between the community composition (at both genus and ASV levels) and environmental parameters was explored by redundancy analysis (RDA) based on the stepwise selection of significant environmental factors (Akaike information criterion, 999 permutations). Distributions of the abundant ASVs (>0.1% of relative abundance) were illustrated by heatmap, with the ASVs clustered by the correlation-based Ward’s hierarchical agglomerative method (52, 53) to identify their distribution patterns and potential ecological partitioning.

### Data availability.

Demultiplexed raw sequence data and metadata were deposited in NCBI as part of BioProject PRJNA794046. Other ancillary data can be found in the GitHub repository (https://github.com/ndxie/EIO-Laby).
