# Supplementary material for: Patchy Blooms and Multifarious Ecotypes of Labyrinthulomycetes Protists and Their Implication in Vertical Carbon Export in the Pelagic Eastern Indian Ocean
Source: Microbiol Spectr. 2022 May 3;10(3):e00144-22. doi: 10.1128/spectrum.00144-22 (PMC9241719; doi:10.1128/spectrum.00144-22)
Supplement: SUPPLEMENTAL FILE 1 — Supplemental material. Download spectrum.00144-22-s001.pdf, PDF file, 0.4 MB [file spectrum.00144-22-s001.pdf]

Patchy blooms and multifarious ecotypes of Labyrinthulomycetes protists and their implication in vertical carbon export in the pelagic Eastern Indian Ocean

Ningdong Xie[1], Mohan Bai[1], Lu Liu[1], Jiaqian Li[1], Yaodong He[1], Jackie L. Collier[3], Dana E. Hunt[4,5], Zackary I. Johnson[4,5], Nianzhi Jiao[1,6], Guangyi Wang[1,2]*

[1]Center for Marine Environmental Ecology, School of Environmental Science and Engineering, Tianjin University, Tianjin 300072, China

[2]Key Laboratory of Systems Bioengineering (Ministry of Education), Tianjin University, Tianjin 300072, China

[3]School of Marine and Atmospheric Sciences, Stony Brook University, Stony Brook, New York 11794-5000, USA

[4]Marine Laboratory, Duke University, Beaufort NC 28516, USA

[5]Biology Department, Duke University, Durham NC 27708, USA

[6]State Key Laboratory of Marine Environmental Science, College of Ocean and Earth Sciences, Xiamen University, Xiamen, Fujian 361005, China

Running title: Labyrinthulomycetes community structure in pelagic waters

Keywords: heterotrophic protist; dark ocean; carbon cycle; biological pump; ecotype.

*** Corresponding author:**

E-mail: gywang@tju.edu.cn

Table S1. Pearson correlations between the Labyrinthulomycetes and prokaryotic cell abundances and environmental parameters.

Environmental parameters	Labyrinthulomycetes abundance			Prokaryotic abundance		
	R	P	N	R	P	N
Depth	-0.115	0.170	144	**-0.309**	**0.000**	139
Temperature	**0.165**	**0.048**	144	**0.579**	**0.000**	139
Salinity	0.034	0.687	144	**-0.627**	**0.000**	139
Density	-0.149	0.074	144	**-0.666**	**0.000**	139
Oxygen	**0.193**	**0.021**	144	**0.607**	**0.000**	139
pH	0.134	0.110	144	**0.493**	**0.000**	139
NH_4	0.077	0.373	137	0.027	0.758	133
SiO_4	-0.151	0.078	137	**-0.261**	**0.002**	133
PO_3	-0.100	0.295	111	**-0.315**	**0.001**	109
NO_3	-0.030	0.751	111	**0.258**	**0.007**	109
NO_2	**0.215**	**0.012**	137	-0.038	0.667	133
TP	**-0.178**	**0.038**	137	**-0.274**	**0.001**	133
TN	-0.148	0.084	137	0.169	0.052	133
Chlorophyll	0.065	0.436	144	0.070	0.416	139

Table S2. Relative abundance and ASV number of each taxonomic group at the genus level.

Taxonomic group (genus level)	Relative abundance	ASV number
Aplanochytrium	47.74%	234
Aurantiochytrium	28.87%	170
Unclassified Labyrinthulaceae	17.16%	163
Unclassified Thraustochytriaceae	2.92%	56
Unclassified Labyrinthulomycetes	1.81%	33
Oblongichytrium	1.20%	12
Thraustochytrium	0.13%	4
Schizochytrium	0.13%	2
Ulkenia	0.04%	2

Table S3. Pearson correlations between the α-diversity indexes of Labyrinthulomycetes and environmental parameters.

Environmental parameters	ASV richness			Community evenness			Shannon diversity		
	R	P	N	R	P	N	R	P	N
Depth	**-0.400**	**0.000**	89	0.155	0.148	89	-0.120	0.261	89
Temperature	**0.498**	**0.000**	89	**-0.368**	**0.000**	89	0.016	0.884	89
Salinity	**-0.235**	**0.027**	89	0.175	0.101	89	-0.006	0.955	89
Oxygen	**0.308**	**0.003**	89	**-0.455**	**0.000**	89	-0.173	0.104	89
pH	**0.441**	**0.000**	89	**-0.249**	**0.019**	89	0.034	0.754	89
Density	**-0.475**	**0.000**	89	**0.390**	**0.000**	89	0.015	0.888	89
Chlorophyll	**0.281**	**0.008**	89	-0.147	0.170	89	0.047	0.663	89
NH_4	0.026	0.833	70	0.039	0.749	70	0.036	0.766	70
SiO_4	**-0.342**	**0.004**	70	0.165	0.173	70	-0.080	0.510	70
PO_3	**-0.392**	**0.002**	59	**0.425**	**0.001**	59	0.096	0.469	59
NO_3	-0.116	0.381	59	-0.115	0.386	59	-0.166	0.209	59
NO_2	-0.095	0.436	70	0.185	0.125	70	0.095	0.432	70
TP	-0.150	0.216	70	0.138	0.255	70	0.035	0.773	70
TN	-0.040	0.743	70	0.165	0.174	70	0.081	0.505	70
Prokaryotic abundance	0.232	0.055	69	**-0.308**	**0.010**	69	-0.097	0.428	69
Labyrinthulomycetes abundance	-0.189	0.114	71	**-0.342**	**0.004**	71	**-0.382**	**0.001**	71

Table S4 Significance (FDR adjusted P-values) of pairwise PERMANOVA tests for the community dissimilarity between different water layers.

	5 m	25 m	50 m	75 m	100 m	200 m	300 m	500 m	1000 m
25 m	0.306	-	-	-	-	-	-	-	-
50 m	0.470	0.506	-	-	-	-	-	-	-
75 m	**0.012**	0.086	0.575	-	-	-	-	-	-
100 m	**0.008**	**0.006**	0.477	0.326	-	-	-	-	-
200 m	**0.010**	**0.013**	0.506	**0.010**	0.125	-	-	-	-
300 m	**0.047**	0.064	0.455	0.064	0.183	0.735	-	-	-
500 m	**0.006**	**0.008**	0.052	**0.006**	**0.010**	0.062	0.914	-	-
1000 m	**0.006**	**0.006**	0.102	**0.006**	**0.030**	0.306	0.914	0.756	-
2000 m	**0.008**	**0.006**	0.064	**0.006**	**0.013**	0.125	0.791	0.521	0.686

Table S5. Stepwise selection based on the RDA model at the ASV level.

Step	RDA model factor	Df	AIC	F	Pr（> F）
0	None		472.56		
1	+ Temperature	1	464.68	10.2079	0.005
2	+ Salinity	1	462.97	3.6595	0.005
3	+ Oxygen	1	461.57	3.3077	0.005
4	+ Chlorophyll	1	461.40	2.0815	0.005

Table S6. Stepwise selection based on the RDA model at the genus level.

Step	RDA model factor	Df	AIC	F	Pr（> F）
0	None		302.29		
1	+ Temperature	1	277.48	30.5859	0.005
2	+ Density	1	273.38	6.0935	0.005
3	+ Salinity	1	272.40	2.8980	0.025
4	+ Depth	1	268.87	5.3887	0.005

Table S7. Spearman correlations between depth and environmental parameters that contained no missing values for the sequenced samples.

Environmental parameters	Spearman ρ	P	N
Temperature	-0.976	0.000	89
Salinity	0.462	0.000	89
Oxygen	-0.646	0.000	89
pH	-0.809	0.000	89
Density	0.977	0.000	89
Chlorophyll	-0.361	0.001	89

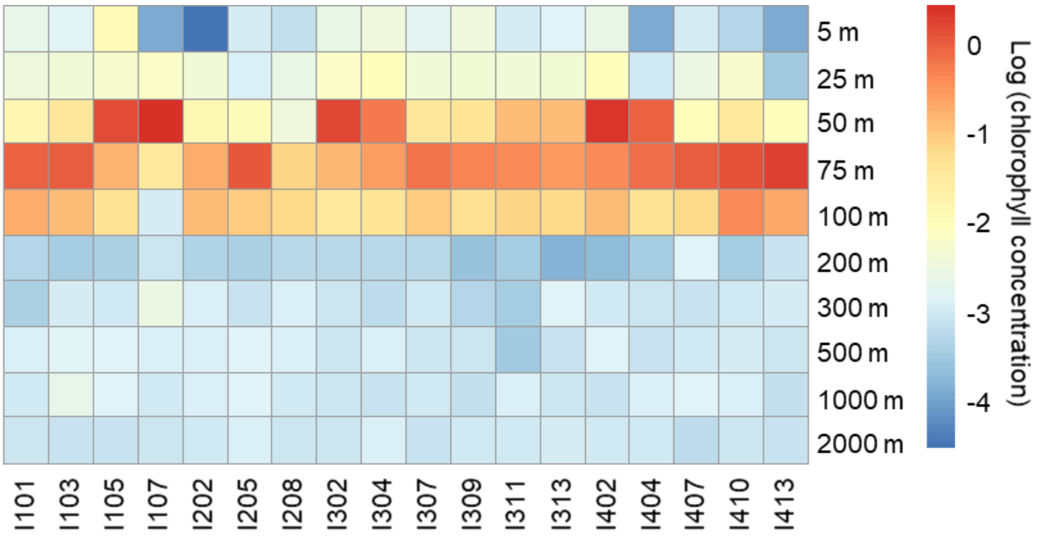

Figure S1. Spatial patterns of chlorophyll concentrations across stations and depths.

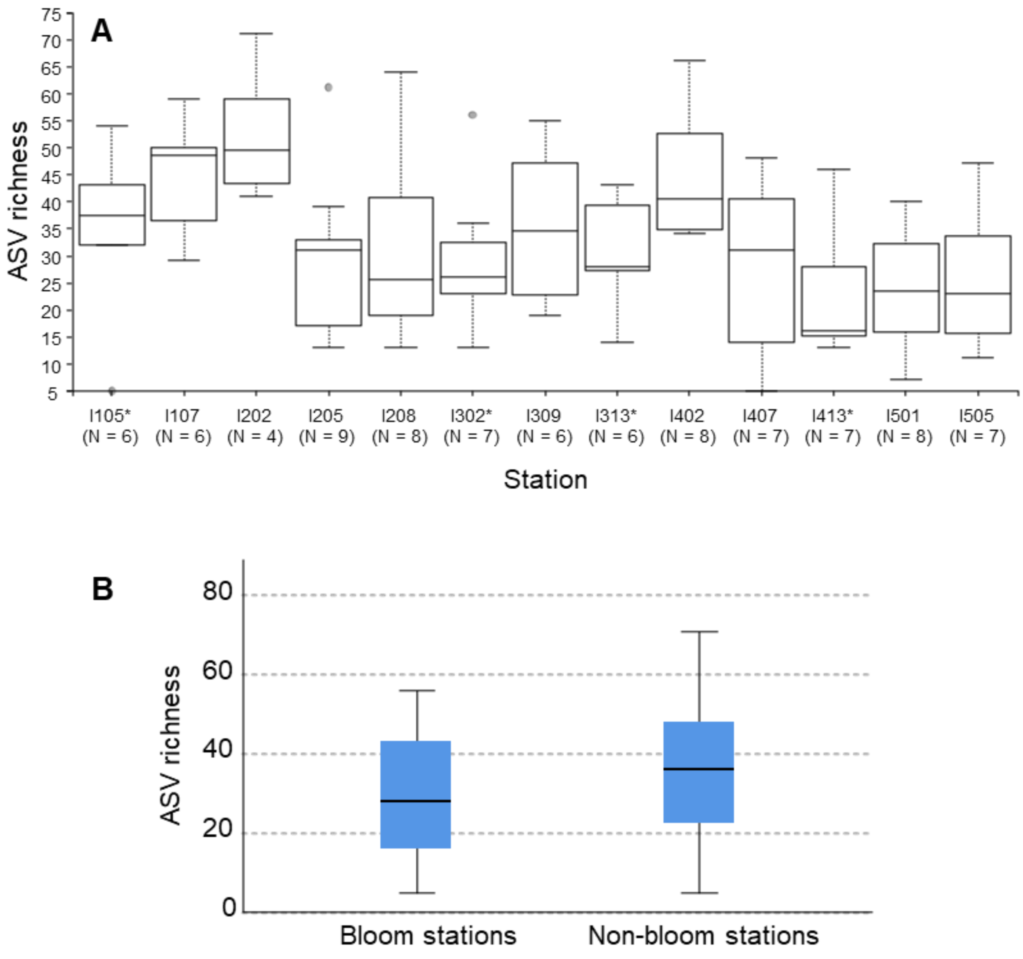

Figure S2. Spatial patterns in Labyrinthulomycetes ASV richness across each station (A) and between the bloom and non-bloom stations (B) in the pelagic waters of the Eastern Indian Ocean. The bloom stations (as defined in Figure 2) were marked with asterisks and the remaining stations except I501 and I505 (lacking cell abundance data) were non-bloom stations.

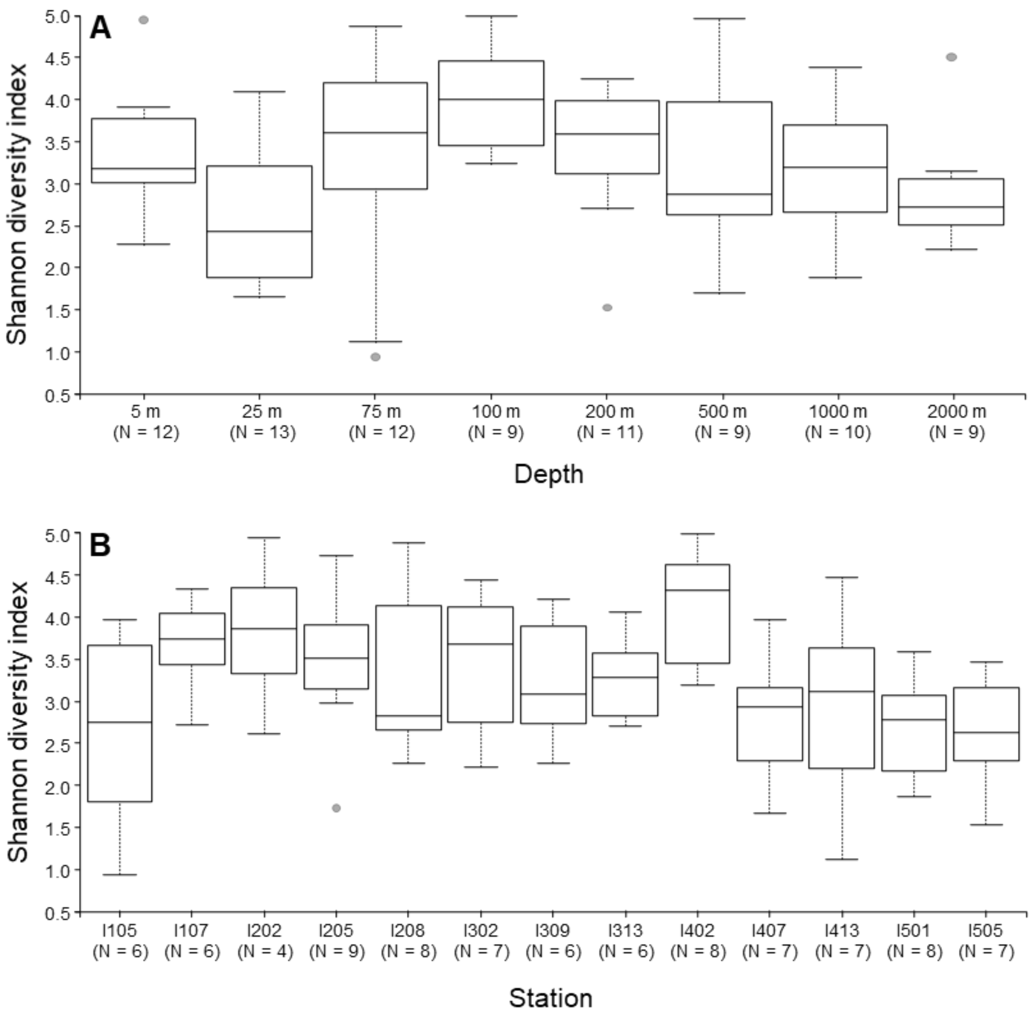

Figure S3. Spatial patterns in Shannon's diversity of Labyrinthulomycetes protists across depths (A) and stations (B) in the pelagic waters of the Eastern Indian Ocean. Groups with small sample size (50 m and 300 m, N < 3) are excluded from the boxplots.

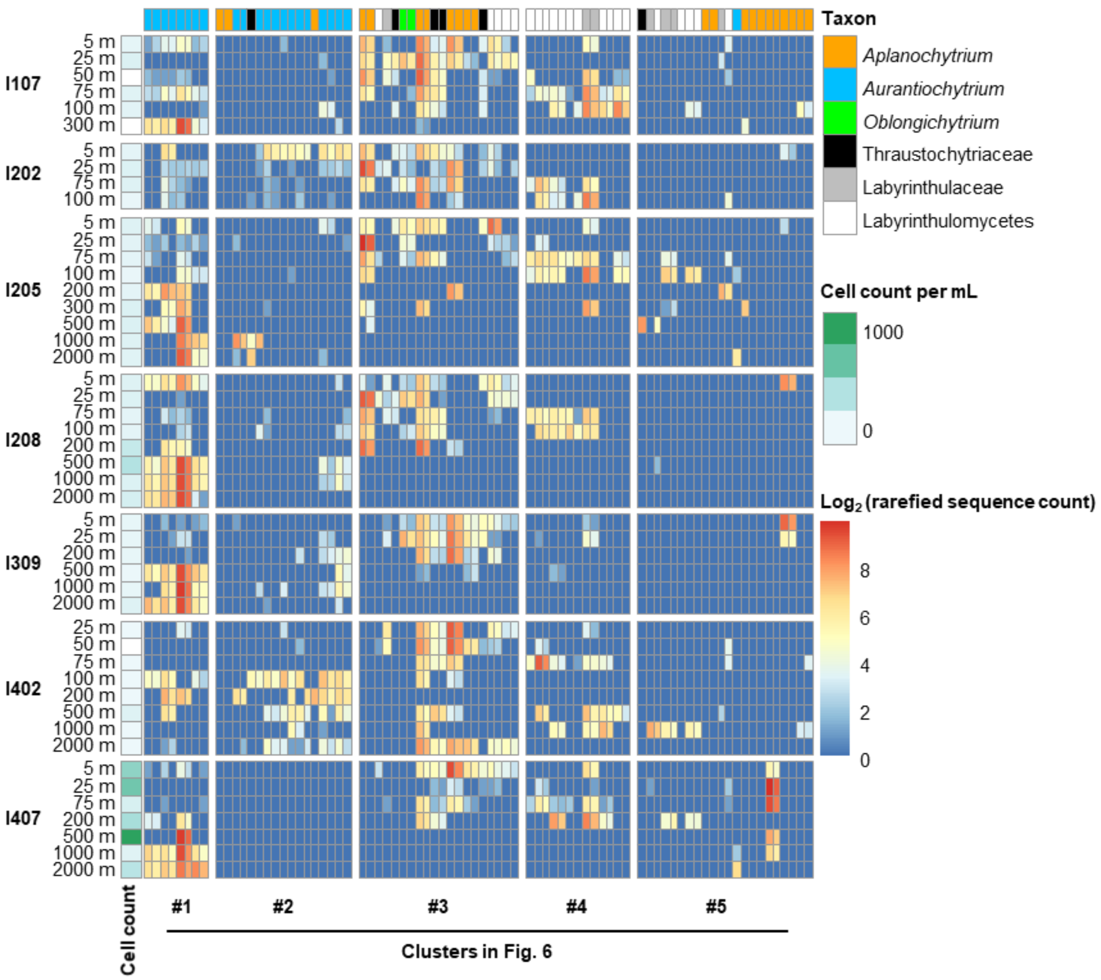

Figure S4. Heatmap showing the distributions of the 80 abundant Labyrinthulomycetes ASVs (whose relative abundance was higher than 0.1% in the total samples) at the non-bloom stations as a comparative reference for Figure 7. Samples (rows) were grouped by stations and then ordered by depth for each station, and were annotated with the total Labyrinthulomycetes cell abundance. ASVs (columns) were arranged by the consistent order of Figures 6 and 7, and were annotated with their taxonomic groups at the genus or the most specific classified level.
